# E-learning in a Jordanian higher education institution

**DOI:** 10.3389/fpsyg.2023.1136142

**Published:** 2023-06-06

**Authors:** Areen Alnemrat, Hesham Aldamen, Mutasim Al-Deaibes, Rami Alsharefeen

**Affiliations:** ^1^Department of Curricula and Instruction, English and Literature, Yarmouk University, Irbid, Jordan; ^2^Department of English, Khalifa University, Abu Dhabi, United Arab Emirates; ^3^Department of Faculty Affairs, Rabdan Academy, Abu Dhabi, United Arab Emirates

**Keywords:** e-learning, faculty attitudes, higher education, Jordanian higher education, online learning

## Abstract

This study seeks to understand the current level of e-learning and to investigate the challenges to the successful implementation of e-learning at a major Jordanian higher education institution from the perspectives of faculty members. Analyses of emailed survey data from 157 faculty members showed that the level of faculty knowledge of e-learning was good (*M* = 3.049) on the 4-point Likert scale. The usage of e-learning by the faculty members was often (*M* = 3.640) on the 5-point Likert scale. Ratings of the policy and support barriers indicated that Yarmouk University faculty members benefit from the technical support that their departments offer to implement e-learning, but the overall responses to the policy and support barriers were undecided (*M* = 3.567). Also, overall Yarmouk University faculty members’ responses to the infrastructure and resources barriers were undecided (3.482). Attitude item responses showed that Yarmouk University faculty members have positive attitudes and a willingness to implement e-learning in their teaching (*M* = 3.913). Also, responses showed a degree of satisfaction of faculty members with the development plans and strategies associated with e-learning (*M* = 3.668). They showed that they did not have obstacles in preparation and development and that they benefited from their plans and strategies. The results showed that there were no differences between males and females on e-learning knowledge, usage, and barriers.

## 1. Introduction

E-learning (learning *via* electronic media) is “a learning technology system that employs web browsers as the major method of interaction with learners and the internet or an intranet as the primary means of communication among its subsystems and with other systems.” These systems serve as a platform for teaching and learning” (Ngai et al., 2007, p. 252). According to Al Suwailem (2018), e-learning is a type of electronically distributed guidance that promotes learning through resources such as web-based workshops, web tutorials, discussion boards, online reviews, and more. Researchers examined e-learning from the widest to the narrowest definition in a study, and it was first any learning that is electronically enabled. Then there is learning, which is made possible by the use of digital technologies. And in the last one, it becomes any internet-enabled or web-based learning (Abbad et al., 2009). Some academics describe e-learning as “communication and learning activities using computers and networks (or *via* electronic methods)” (Sambrook, 2003; OECD, 2005).

E-learning allows students to learn regardless of their physical location or the distance between them and the learning environment. According to Almarashdeh et al. (2011), e-learning platforms provide teachers with a platform for creating and delivering course material, assessing student performance, and monitoring learner participation. Self-paced instruction, asynchronous facilitator-led instruction in which students and teachers interact at different times, and synchronous facilitator-led instruction in which students and teachers interact at the same time are all examples of e-learning. Self-paced and facilitator-led modules are being actively integrated by e-learning developers to provide an immersive experience (Mason, 2006). According to one research, e-learning has become a more social platform as a result of interactions between students and professors, or students and students, utilizing various learning technologies (Asmaa and Najib, 2016). According to Alwi and Fan (2010), e-learning resources provide learners with the capacity to exchange knowledge in multiple media, such as slideshows, video, and PDFs, as well as communicate with the teacher through chatting in online courses. E-learning may also assist graduates develop more skills than traditional higher education students (El-Gamal, 2014). Concannon and Campbell (2005) shown in their study that e-learning might provide various benefits, including access to educational materials, which is especially important when some libraries are short on books. E-learning also provides the ability to search for information in a central facility that provides access to comprehensive sources of all academic resources.

Information technology has evolved into a way of life, rather than a luxury tool for a specific industry or social elite. The global trend toward knowledge economies is based on the use of modern technologies to invest knowledge in improving social welfare and diversifying resources. As a criterion for progress and prosperity, information technology has become an indispensable means of survival in an open and competitive world. Emerging generations are always capable of making a qualitative leap if given the opportunity to do so. The educational system is the most important engine for radical change and a true revolution in lifestyle and thinking in this context.

According to studies, e-learning systems are extremely beneficial because they provide flexibility and continuous access to educational materials (Bostrom, 2003; Parker, 2003). E-learning courses are becoming an increasingly important part of higher education (Ngai et al., 2007). E-learning also provides the ability to search for information in a single location, facilitating access to comprehensive sources of all scientific materials (Concannon and Campbell, 2005). E-learners can then access learning resources at any time and from any location, resulting in increased learning productivity and efficiency for students (Vidyashree and Kumar, 2016).

E-learning has recently emerged as an important agenda item in higher education in order to provide broad-ranging, long-term training to a diverse audience by creating, delivering, and managing course content electronically (Mason, 2006). E-learning systems offer numerous features to teachers and students in order to save time and money by removing the need for separation (Ayu, 2020). Despite all that e-learning has to offer instructors, students, and classrooms, some academic systems are still in their early stages of implementation. This is true of Jordan’s higher education institutions. To date, Jordanian schools and universities have very limited access to e-learning systems; they continue to use the traditional method of instruction based on in-person interactions between teachers and students (Alkhawaja and Halim, 2019).

The importance of the study stems from the fact that it provides new insights into the use of e-learning in Jordanian universities in general and during the he novel coronavirus outbreak. It also attempts to identify the e-learning challenges that faculty members face in higher education. The findings provide feedback to Jordanian university policymakers in order to expose the e-learning issues that faculty members face. Diagnosing e-learning challenges assists university faculty members and administrators in confronting and addressing these challenges by modifying, developing, and/or adopting new methods of teacher training and preparation programs for effective implementation of the Jordanian e-learning system.

The study seeks answers to the following research questions. Those answers will provide an empirical general foundation of three major aspects (knowledge, usage, and obstacles) of embracing e-learning in higher education. This information will assist decision makers and investors in taking actions toward successful e-learning in light of the weaknesses and strengths shown by these research questions.

1. What do the academic members at Yarmouk University know about e-learning?
2. To what degree do faculty members at Yarmouk University include e-learning into their classroom instruction?
3. What are the biggest barriers that Yarmouk University faculty members may face in appropriately incorporating e-learning into their teaching?
4. Is there a difference between male and female Yarmouk University faculty members in:
  - their comprehension of e-learning;
  - their utilization of e-learning; and
  - the difficulties they face?
5. Is there correlation between faculty members’ selected demographic factors (gender, academic department, years of teaching experience, degree of technical skills, technology-training programs, and internet access) and adoption of e-learning?

## 2. Literature review

### 2.1. E-learning and gender differences

There has been various research that have revealed the existence and implications of gender disparities in e-learning. These studies are frequently contradictory. Shaw and Gant (2002) indicate in their study that the gender difference is closing with time. Another research found that men and women go through different stages of tolerance, worry, and curiosity when it comes to modern technologies (McCoy and Heafner, 2004). Cuadrado-García et al. (2010) investigated the presence of significant differences in the use of male and female e-learning and assessment practices in an online project and discovered that there are few differences between male and female students in their use of e-learning as well as their motivation and satisfaction.

Al Suwailem (2018) conducted a study that found gender had no significant influence on faculty members’ intent to employ the e-learning approach at one Saudi Arabian university. Al-Emran and Shaalan (2015), on the other hand, concluded that female educators’ attitudes toward mobile learning was more positive than male educators’ attitudes in one of the developing countries.

### 2.2. Adoption of e-learning in higher education

Qteishat and Alshibly (2013) carried out research in Jordan that found students have a key role in the adoption of e-learning. According to the findings of this study, students’ attitudes about e-learning can be a significant element in the adoption of e-learning in higher education. According to the findings of this study, those who have a good attitude toward e-learning have a higher intention to accept the system and are thus more likely to utilize it.

A similar study was conducted in Libya by Benghet and Helfert (2014). They investigated the variables influencing the acceptability of e-learning adoption in higher education and discovered a set of criteria that might play an essential influence in e-learning adoption and acceptance. These variables were divided into three categories: organizational impediments, technological barriers, and social constraints. Organizational hurdles relate to the settings and preparations that institutions should consider in order to promote adoption acceptability, such as increased workload for educational personnel and a lack of support for educational elements of advancement. There are also a number of challenges grouped as technological hurdles, but the issue is that many students and staff members do not have access to a personal computer or the internet. Social barriers arise when a group is unable to acknowledge or accept a new process in a crucial aspect of their life owing to factors such as spiritual ideals, social taxes, or habits.

A case study in Northern Iraq attempted to investigate the elements for effective e-learning adoption from the providers’ and students’ perspectives in private colleges and concluded that cultural acceptability is a critical component for having sustained e-learning applications (Abdullah et al., 2017).

In Saudi Arabia, a group of researchers conducted a study based on the Technology Acceptance Model (TAM) to explore the specific factors affecting successful implementation of e-learning in higher education and to examine whether the e-learning is reliable, effective, easy-to-use, and cost-effective at the higher education sector, and they found that self-efficacy, compatibility, facilitating conditions, training, and gender are important elements that effect adoption of e-learning (Solangi et al., 2018).

The success rate of delivery through virtual teaching varies from institution to institution (Crawford et al., 2020) and the level of success in which institutions transitioned to the online mode was not only impacted by internet access and reliability, but also with faculty competence in online pedagogy, institution adoption of online systems, and faculty and student prior experience with online learning (Sasere and Makhasane, 2020). A rate of over 80% of higher education institutions in developed countries switched to virtual/online lecture delivery and assessment. In developing economies, which Jordan is part of, over 80% of the Higher education institutions partially switched to virtual education and some were not able to move fully to online (Crawford et al., 2020, p. 21).

Studies in comparable milieus were conducted and reported on successes and challenges associated with student and faculty adoption of online learning (see Adnan and Anwar, 2020; Davies and Al Sharefeen, 2022, 2022a).

### 2.3. E-learning in Jordan

Jordan prioritizes education in general, and higher education in particular, believing that it is a worthwhile investment. Jordan has a human capital of university students numbering in the thousands, and these students are expected to be academically and technologically literate, as well as equipped for life, in order to boost the country’s economy through technology. The Ministry of Education has launched a national e-learning plan through knowledge networks across the country in partnership with the Ministries of Planning and Information and Communication Technology. Instead of the traditional teacher-led learning system, information and communication technology is employed as the foundation of a learning framework centered on the development of self-learning and critical thinking. Similarly, Jordanian institutions have established e-learning programs targeted at increasing the efficacy and efficiency of education for both teachers and students (Ministry of Higher Education and Scientific Research, 2021).

Some academics explored various issues of e-learning in Jordanian higher education at the empirical evidence level (Al-Jedaiah, 2020). One of these studies focused on the problems of e-learning for faculty members in Jordanian private universities. This study identified and categorized the faculty members’ identified difficulties, ranking them from the most pressing to the least important (maximum to minimum). These difficulties were: academic research problems through the Internet (medium level); professional obstacles for employing e-learning techniques (medium level); and university financial and administrative challenges (medium degree). The three most pressing challenges in the field of academic research challenges *via* the Internet were proficiency in the English language, dealing with peer-reviewed electronic journals for research and publication, and keeping up with new academic publications and modern software in e-learning. The most pressing obstacles in the field of professional challenges for using e-learning techniques were the heavy teaching load of faculty members, the lack of e-learning courses and training programs for faculty members, and time constraints associated with preparing and developing e-learning programs. Finally, the most pressing obstacles in the field of university financial and administrative challenges were the availability of an adequate number of computers connected to the Internet, the high financial cost of e-learning software and equipment, and the technological infrastructure to provide and succeed e-learning programs (Al-Qudah and Maqableh, 2014).

### 2.4. Higher education in Jordan

The Hashemite Kingdom of Jordan’s Ministry of Higher Education and Scientific Research (2021) discusses higher education levels in depth on its official website. Students who have completed a regular high school diploma are eligible to enroll in Jordanian higher education. Jordan has both public and private postsecondary education institutions, which include community colleges and universities.

#### 2.4.1. University education in Jordan

Jordan’s higher education system began in 1951, with the establishment of several teacher colleges. Jordan’s University was established in 1962. In 1976, Yarmouk University and six other public institutions in other locations of the nation were created. Amman University was Jordan’s first private university, founded in 1990, and numerous more Jordanian private institutions have subsequently been formed. Jordan now has ten governmental universities and 19 private institutions.

#### 2.4.2. Non-university postsecondary education

Jordan has community colleges that provide non-university postsecondary education. Jordan launched its first community colleges in 1981. This sort of institutions provides advanced preparation as well as career-oriented preparation and prepares students for intermediate skilled job. Jordanian community colleges provide two-year intermediate research courses in the following fields: architecture, technical studies, meteorology, administration, social work, hotel management, IP, paramedic science, agriculture, training, and liberal arts.

### 2.5. Yarmouk University

Yarmouk University was chosen as the locale for this study. Yarmouk University was established in 1976. The institution has developed and expanded, transforming it into a community with several schools and services. In each of its 15 faculties, Yarmouk University provides 63 master’s degrees, 19 doctoral programs, and bachelor’s degree programs. In addition, the institution offers 11 research and professional development programs. The institution now has 27,850 students, 1,012 faculty members, and 1,740 administrative and technical staff members.

## 3. Methodology

A survey was utilized to assess e-learning understanding, utilization, and faculty members’ perspectives on challenges associated with e-learning. The demographic information is used to derive the independent variables, which include gender, academic department, years of teaching experience, technological skill level, technology training programs, and internet access. The dependent variables in this study are the faculty members’ degree of e-learning expertise, their usage of e-learning, and the difficulties they have encountered. The study included both female and male faculty members who taught at Yarmouk University in the fall of 2020. Members of the faculty have varying degrees of professional abilities and years of teaching experience.

According to the Scientific Research and Graduate Studies Deanship at Yarmouk University (2021), there are 1,012 faculty members in total. This study’s sample was drawn at random from the entire population (faculty members). The Scientific Research and Graduate Studies Deanship (SRGSD) of Yarmouk University extended the email invitation to the target faculty members. The SRGSD contacted 200 faculty members and received responses from 157 individuals. Accordingly, the participant sample for the study was 15% of the faculty members who were working at Yarmouk University during Fall 2020.

### 3.1. Data collection

A survey was utilized to obtain data on the deployment of e-learning in Jordanian higher education. The survey was created after revising a number of existing surveys related to technology studies. Some survey items were written by the researcher, while others came from literature reviews. The majority of the items are from one source and have been updated to meet the needs of this research. This reference was 2015 research titled “Barriers to integrating information technology in Libyan higher education” by Faiza Elshaikhi. To utilize selected items from the Elshaikhi (2015) survey, the researcher obtained permission from Dr. Elshaikhi, who granted authorization to use the survey or specific artifacts. Following the revisions and alterations, this study survey is divided into three major sections. The first section focuses on demographic data. This section has six questions designed to elicit information on gender, academic department, years of teaching experience, technological skills level, technology-training programs, and internet access.

The second component assesses the present degree of e-learning implementation and is divided into two pieces. Section one is meant to collect information regarding e-learning knowledge and consists of nine questions. The responses of faculty members were graded on a four-point Likert scale (1 = Very Little Experience, 2 = Some Experience, 3 = Good Experience, 4 = A Lot of Experience). Section two is for e-learning usage and has seven items. Faculty member responses are assessed on a five Likert-type scale of (1 = Never, 2 = Rarely, 3 = Sometimes, 4 = Often, 5 = Always).

The third section, which has four elements, calculates the implementation hurdles. Responses from faculty members are scored on a five-point Likert scale (1 = Strongly Disagree, 2 = Disagree, 3 = Undecided, 4 = Agree, 5 = Strongly Agree). Part one, which consists of seven topics, is about policy and support. The second part, which consists of seven issues, is on infrastructure and resources. The third part, which consists of eight subjects, is on faculty members’ attitudes about e-learning. The final component, which consists of six elements, is about planning and development.

The Cronbach Alpha test was used to ensure the reliability of the questions asked in the questionnaire to test the study questions and answer them for the purposes of testing the reliability of the questionnaire, as this percentage must be greater than 60% to be acceptable for the purposes of scientific research (Collis and Hussey, 2013). The Cronbach Alpha test results are shown in Table 1.

**Table 1:** Reliability test.

Parts	No of items	Cronbach’s alpha
Knowledge of E-learning	9	0.873
Usage of E-Learning	7	0.892
*Part II: Current level of implementation of E-learning*	**16**	**0.926**
Policy and support	7	0.812
Infrastructure and resources	7	0.849
Attitudes of faculty members about implementation of E-LEARNING	8	0.847
Preparation and development	6	0.825
*Part III: The implementation of E-learning*	**28**	**0.929**

The previous table shows that the alpha coefficient values varied between (81.2 and 95%), indicating a good stability factor.

### 3.2. Statistical analysis

Following data collection, the researcher utilized the (SPSS) Statistical Package for Social Science to analyze the study questions, and all analyses were done at the statistical significance level *α* = 0.05. The researchers employed descriptive statistics to answer the first three questions, which provide data on the mean, standard deviation, frequency, variance, range, and percentage of individuals answering for each group. To address the fourth question, the researcher used an independent samples *t*-test to evaluate the gaps in e-learning understanding, e-learning usage, and obstacles between male and female faculty at Yarmouk University. To answer the fifth question, the researcher used multiple regression to evaluate how demographic factors of faculty members may predict the utilization of e-learning in their teaching.

## 4. Results

### 4.1. Description of population and sampling

The questionnaire’s sample comprises both male and female faculty members from Jordan’s Yarmouk University. The survey was emailed to 200 faculty members in the fall of 2020, and 157 responded (Table 2).

**Table 2:** Distribution of the study sample by gender.

Type of gender	Frequency	Percent
Male	83	52.9
Female	74	47.1
Total	157	100

The number of female respondents was 74, representing 47.1% of the study sample, while the number of male respondents was 83, representing 52.9% of the study sample.

Table 3 displays the number of years of teaching experience for the study sample, revealing that 43.3% of the study sample had 3–6 years of teaching experience at the University (19.1% male and 24.2% female), with 24.9% of faculty members having more than 10 years of experience (20.4% male and 4.5% female). Finally, 10.8% of faculty members had less than 3 years of experience (9.6% were male and 11.4% were female), and 21% had 7–10 years of experience (3.8% male and 7% female).

**Table 3:** Distribution of the study sample by years have been teaching.

How many years have you been teaching?	Gender	Frequency	Percent
Less than 3 years	Male	6	3.8
	Female	11	7.0
3–6 years	Male	30	19.1
	Female	38	24.2
7–10 years	Male	15	9.6
	Female	18	11.4
More than 10	Male	32	20.4
	Female	7	4.5
Total	Male	83	52.9
	Female	74	47.1

Table 4 shows the level of technology skill of the faculty members, which shows that 66.2% of the faculty members have intermediate skills in technology (34.5% male and 31.7% female) and 29.9% of the faculty members have advanced skills in technology (15.9% male and 14% female), while 3.8% of the teaching staff has beginner level skills (2.5% male and 1.3% female).

**Table 4:** Distribution of the study sample by technological skills level.

What is your level of technology skills?	Gender	Frequency	Percent
Beginner	Male	4	2.5
	Female	2	1.3
Intermediate	Male	54	34.5
	Female	50	31.7
Advanced	Male	25	15.9
	Female	22	14.0
Total	Male	83	52.9
	Female	74	47.1

Table 5 shows when faculty members obtained technology training programs, with 50.3% obtaining in-service and pre-service technology training programs (22.3% male and 28% female), 26.1% obtaining pre-service technology training programs (16.6% male and 9.5% female), and 12.1% obtaining in-service technology training programs (7% male and 4.5% female).

**Table 5:** Distribution of the study sample by obtained the technology-training.

When do you obtain the technology-training program?	Gender	Frequency	Percent
Pre-service	Male	26	16.6
	Female	15	9.5
In-service	Male	11	7.0
	Female	8	5.1
Pre-service and in-service	Male	35	22.3
	Female	44	28.0
None	Male	11	7.0
	Female	7	4.5
Total	Male	83	52.9
	Female	74	47.1

Table 6 shows the major to which faculty members belong, and it shows that the highest percentage of respondents (24.2%) represented faculty members in the Faculty of Economics (11.5% male and 12.7% female), while the lowest percentage (0.6% male and 0% female) represented faculty members in the Faculty of Computer Science.

**Table 6:** Distribution of the study sample by major.

Major	Gender	Frequency	Percent
Faculty of Arts	Male	7	4.5
	Female	5	3.2
Faculty of Economic	Male	18	11.5
	Female	20	12.7
Faculty of Anthropology	Male	2	1.3
	Female	0	0.0
Faculty of Education	Male	7	4.5
	Female	8	5.1
Faculty of Sport	Male	7	4.5
	Female	4	2.5
Faculty of Hotel	Male	2	1.3
	Female	4	2.5
Faculty of Sharia	Male	14	8.9
	Female	13	8.3
Faculty of Pharmacy	Male	2	1.3
	Female	0	0.0
Faculty of Medicine	Male	0	0.0
	Female	2	1.3
Faculty of Science	Male	17	10.8
	Female	15	9.6
Faculty of Fine Arts	Male	2	1.3
	Female	2	1.3
Faculty of Law	Male	2	1.3
	Female	0	0.0
Faculty of Engineering	Male	2	1.3
	Female	1	0.6
Faculty of Computer Science	Male	1	0.6
	Female	0	0.0
Total	Male	83	52.9
	Female	74	47.1

While none of the sample participants responded (No-Access), the greatest frequency of Internet access was 90.4 (47.7% male and 42.7% female), and the rate of Internet access through school was 9.6% (5.1% male and 4.5% female; Table 7).

**Table 7:** Distribution of the study sample by internet access.

Internet access	Gender	Frequency	Percent
At-Home	Male	75	47.7
	Female	67	42.7
At-School	Male	8	5.1
	Female	7	4.5
No-Access	Male	0	0
	Female	0	0
Total	Male	83	52.9
	Female	74	47.1

### 4.2. Findings of the research questions

The Statistical Analysis for Social Sciences Edition 24 program was used to test the study questions as follows.

#### 4.2.1. First question

This section will explain the first question, “What do faculty members at Yarmouk University know about e-learning?” The answers to the first question are shown in Table 8.

**Table 8:** Faculty members at Yarmouk University knowledge about e-learning.

Item No.	Paragraph	Mean	Std. Deviation
1	Distance education in general	2.898	0.700
2	Computerized electronic learning	3.006	0.712
3	online learning	2.981	0.764
4	Internet learning	3.083	0.679
5	Online computing accounts (e.g., Blackboard, WebCT, Vista, …, etc.)	2.694	0.931
6	E-mail programs (e.g., Outlook Express, Yahoo, Hotmail, …, etc.)	3.217	0.728
7	Web page creation programs (e.g., Front Page, Dream weaver)	2.675	0.995
8	Web searching (e.g., Google, Yahoo, etc.).	3.459	0.655
9	Online social networking service (e.g., Facebook, Twitter, etc.)	3.427	0.681
Knowledge of e-learning		3.049	0.541

As indicated in Table 8, the mean of Yarmouk university faculty members’ Knowledge of e-learning achieved 3.049 with a degree of satisfactory experiences. The greatest mean value was for “Web searching (e.g., Google, Yahoo, etc.)” with a value of 3.459, while the lowest mean value was for “Web page construction programs (e.g., Front Page, Dream Weaver)” with a value of 2.675. In general, the results reveal that the faculty members of Yarmouk University have strong expertise with e-learning knowledge.

#### 4.2.2. Second question

This section seeks to provide a solution to the second question, “To what degree do Yarmouk University faculty members employ e-learning in their teaching?” The outcomes of answering the second question are shown in Table 9.

**Table 9:** Faculty members at Yarmouk University extent of using e-learning.

Item No.	Paragraph	Mean	Std. Deviation
1	Update your computer with necessary hardware and software requirements for e-learning.	3.592	0.987
2	Activate your computing accounts, such as Blackboard, Vista, WebCT.	3.357	1.149
3	Manage my courses (e.g., blackboard: post homework or other class requirements, grades, project information or suggestions.)	3.713	1.007
4	Share my students’ work on the Web.	3.586	0.994
5	Support learning and research (e.g., use content-specific tools).	3.688	0.905
6	Collaborate with colleagues and experts/other professionals.	3.803	0.851
7	Communicate with students through the platform outside of classroom hours.	3.739	0.863
Using of e-learning		3.640	0.756

As indicated in Table 9, the mean frequency of use of e-learning by Yarmouk University faculty members was 3.640. The item with the greatest average was “Collaborate with colleagues and experts / other professionals” with a value of 3.803, while the one with the lowest average was “Activate your computer accounts, such as Blackboard, Vista, WebCT” with a value of 3.357. On average, the data suggest that Yarmouk University faculty members frequently use e-learning.

#### 4.2.3. Third question

This section seeks to address the third question, “What are the primary challenges that may prohibit faculty members at Yarmouk University from properly using e-learning in their teaching?” This question has been broken into three parts, as follows:

To examine the issue, “What are the main policy and support impediments that can prohibit faculty members at Yarmouk University from effectively using e-learning in their teaching?” The outcomes of answering this question are shown in Table 10.

**Table 10:** The implementation of e-learning (Policy and support).

Item. No.	Paragraph	Mean	Std. Deviation
1	Our university has a good strategic plan for the implementation of e-learning.	3.452	0.916
2	There is a specific budget for e- learning in our university.	3.357	0.817
3	There is obligation from the ministry to let me use e-learning.	3.656	0.838
4	Specialists follow the implementation of e-learning that I use in my teaching.	3.490	0.844
5	There is a tangible motivation from education community to use e-learning.	3.707	0.819
6	There is enough technical support/advice for e-learning in our department.	3.694	0.822
7	The ministry of education does require me to use e-learning in my teaching.	3.611	0.998
Policy and support		3.567	0.594

As indicated in Table 10, the mean of policy and support hurdles that would prohibit Yarmouk University faculty members from implementing e-learning reached 3.567 with a degree of uncertainty. The item with the greatest average was “There is a tangible motivation from the education community to utilize e-learning,” with a value of 3.707, while the item with the lowest average was “There is a dedicated budget for e-learning in our institution,” with a value of 3.357. On average, the results suggest that Yarmouk University faculty members are both neutral and supportive of the policy.

To examine the subject, “What are the primary infrastructure and resource hurdles that can prohibit faculty members at Yarmouk University from efficiently using e-learning in their teaching?” The outcomes of answering this question are shown in Table 11.

**Table 11:** The implementation of e-learning (Infrastructure and resources).

Item. No.	Paragraph	Mean	Std. Deviation
1	There are enough computers and other computer peripherals at our university.	3.379	1.111
2	The architecture of classrooms is suitable enough to use e-learning.	3.471	1.078
3	There is Internet service in my classroom.	3.427	1.128
4	Students do have an opportunity to access the Internet during the school day.	3.605	0.890
5	Internet connection is fast enough for use while teaching.	3.325	1.145
6	There are computerized textbooks for most of our curricula.	3.366	1.157
7	I can access technical support in using e-learning.	3.803	0.788
Infrastructure and resources		3.482	0.761

According to Table 11, the average of the infrastructure and resource hurdles in implementing e-learning among Yarmouk university faculty members is 3.482, with a degree of uncertainty. The greatest average was for the item “I can contact technical help when using e-learning.” with a value of 3.803, while the lowest average was for the item “Internet connection is fast enough for usage during teaching” with a value of 3.325. On average, the results reveal that Yarmouk University’s faculty members are unconcerned about infrastructure and resources.

To explore question three, “What are the primary attitudes that may inhibit faculty members at Yarmouk University from properly using e-learning in their teaching?” The outcomes of answering this question are shown in Table 12.

**Table 12:** The implementation of e-Learning (Attitudes).

Item. No.	Paragraph	Mean	Std. Deviation
1	I believe in the importance of using technology in teaching.	4.000	0.840
2	I am interested in implementing e-learning to deliver courses.	4.013	0.809
3	Our department chair has positive attitudes towards implementing e-learning.	3.796	0.752
4	I believe that using e-learning will improve my teaching skills.	3.911	0.880
5	I think it is easy for me to manage the classroom while applying e-learning.	3.873	0.897
6	I have time to develop the activities/lessons that use information technology.	3.745	0.973
7	Implementing e-learning increases the social interaction between my students and me.	3.847	0.972
8	I am willing to collaborate with specialists to implement e-learning in my teaching.	4.121	0.692
Attitudes		3.913	0.595

According to Table 12, the mean of Yarmouk University faculty members’ attitudes toward e-learning implementation reached 3.913 with a degree of Agree, the highest average was for “I am willing to collaborate with specialists to implement e-learning in my teaching “item with a value of 4.121, and the lowest value of mean was for “I have time to develop activities/lessons that use information technology “item with a value of 3.745. The findings suggest that, on average, Yarmouk University faculty members are open to incorporating e-learning into their classroom instruction.

To examine the issue, “What are the primary preparation and development challenges that can prohibit Yarmouk University faculty members from effectively using e-learning in their teaching?” The outcomes of answering this question are shown in Table 13.

**Table 13:** The implementation of e-learning (Preparation and development).

Rank	Par. No.	Paragraph	Mean	Std. Deviation	Degree of approval
4	1	E-learning training opportunities are available in our university.	3.713	0.825	Agree
6	2	There is a pre-service training about e-learning skills.	3.497	0.972	Undecided
1	3	There is an in-service training about e-learning skills.	3.764	0.885	Agree
5	4	My pre-service training to use e-learning was good.	3.535	1.041	Undecided
2	5	My in-service training to use e-learning was good.	3.752	0.910	Agree
3	6	I have enough time to learn skills of how to implement e-learning.	3.745	0.926	Agree
Preparation and development			3.668	0.678	Agree

As indicated in Table 13, the mean of the (preparation and development) as an e-learning implementation of Yarmouk University faculty members is 3.668 with “Agree.” The item with the highest average was “There is an in-service training about e-learning skills,” with a value of 3.764, while the item with the lowest mean value was “There is a pre-service training about e-learning skills,” with a value of 3.497. The findings demonstrate that, on average, Yarmouk University faculty members have received training and development for using e-learning in the classroom.

#### 4.2.4. Fourth question

This part seeks to answer the fourth question, “Does there exist a difference between male and female faculty members at Yarmouk University in their understanding of e-learning, their usage of e-learning, and the difficulties they face?” This question has been broken into three parts, as follows:

Is there a difference in e-learning knowledge between male and female Yarmouk University faculty members? Table 14 displays the outcomes of answering this question.

**Table 14:** Difference between male and female in their knowledge of e-learning.

Model summary^c^										
Model	*R*	*R* Square	Adjusted *R* square	Std. Error of the estimate	Change statistics					Durbin-Watson
					*R* square change	*F* change	*df*1	*df*2	Sig. *F* change	
1	0.178^a^	0.032	0.025	0.53455	0.032	5.062	1	155	0.026	
2	0.218^b^	0.047	0.035	0.53189	0.016	2.553	1	154	0.112	1.885

ANOVA^d^						
Model		Sum of squares	*df*	Mean square	*F*	Sig.
1	Regression	1.446	1	1.446	5.062	0.026^b^
	Residual	44.290	155	0.286		
	Total	45.737	156			
2	Regression	2.169	2	1.084	3.833	0.024^c^
	Residual	43.568	154	0.283		
	Total	45.737	156			

Coefficients^d^						
Model		Unstandardized coefficients		Standardized coefficients	*t*	Sig.
		*B*	Std. Error	Beta		
1	(Constant)	2.891	0.082		35.152	0.000
	Department	0.027	0.012	0.178	2.250	0.026
2	(Constant)	2.685	0.153		17.607	0.000
	Department	0.028	0.012	0.184	2.336	0.021
	Gender	0.136	0.085	0.126	1.598	0.112

a Predictors: (Constant), Department.

b Predictors: (Constant), Department, gender.

c Dependent Variable: knowledge of e-learning.

d Dependent Variable: knowledge of e-learning.

Table 14 demonstrates that there are no statistically significant differences > 0.05 that reach (0.112) between the averages of the study sample responses regarding knowledge of e-learning according to gender, implying that both male and female faculty members at Yarmouk University have the same knowledge about e-learning.

Table 15 displays the outcomes of answering the question “Is there a difference in the utilization of e-learning among male and female faculty members at Yarmouk University?”

**Table 15:** Difference between male and female in their use of e-learning.

Model summary^c^										
Model	*R*	*R* square	Adjusted *R* square	Std. Error of the estimate	Change statistics					Durbin-Watson
					*R* square change	*F* change	*df*1	*df*2	Sig. *F* change	
1	0.194^a^	0.038	0.031	0.74409	0.038	6.045	1	155	0.015	
2	0.203^b^	0.041	0.029	0.74502	0.004	0.616	1	154	0.434	1.692

ANOVA^d^						
Model		Sum of squares	*df*	Mean square	*F*	Sig.
1	Regression	3.347	1	3.347	6.045	0.015^b^
	Residual	85.820	155	0.554		
	Total	89.167	156			
2	Regression	3.688	2	1.844	3.323	0.039^c^
	Residual	85.478	154	0.555		
	Total	89.167	156			

Coefficients^d^						
Model		Unstandardized coefficients		Standardized coefficients	*t*	Sig.
		*B*	Std. Error	Beta		
1	(Constant)	3.399	0.114		29.694	0.000
	Department	0.042	0.017	0.194	2.459	0.015
2	(Constant)	3.258	0.214		15.251	0.000
	Department	0.042	0.017	0.197	2.491	0.014
	Gender	0.094	0.119	0.062	0.785	0.434

a Predictors: (Constant), department.

b Predictors: (Constant), department, gender.

c Dependent variable: use of e-learning.

d Dependent variable: use of e-learning.

Table 15 demonstrates that there are no statistically significant differences > 0.05 that reach (0.434) between the averages of the study sample responses regarding usage of e-learning by gender, indicating that both male and female faculty members utilize e-learning at Yarmouk University.

The outcomes of answering the question “Is there a difference in the hurdles to e-learning faced by male and female faculty members at Yarmouk University?” are shown in Table 16.

**Table 16:** Difference between male and female in their barriers of e-learning.

Section	Sub-section	Gender	No	Mean	Std. Dev.	*T*	Sig.
Barriers of e-learning implementation	Policy and support	Male	83	3.542	0.591	–0.551	0.583
		Female	74	3.595	0.601		
	Infrastructure and resources	Male	83	3.525	0.777	0.744	0.458
		Female	74	3.434	0.744		
	Attitudes	Male	83	3.895	0.612	–0.415	0.679
		Female	74	3.934	0.579		
	Preparation and development	Male	83	3.637	0.672	–0.609	0.544
		Female	74	3.703	0.689		

Table 16, shows that there are no statistically significant differences > 0.05 between the averages of the study sample responses regarding the barriers of e-learning implementation based on gender, implying that both male and female faculty members face the same e-learning implementation barriers at Yarmouk University.

#### 4.2.5. Fifth question

This section seeks to answer the fifth question, “Are the selected demographic variables of faculty members (gender, academic department, years of teaching experience, technology skills level, technology-training programs, internet access) related to the implementation of e-learning in their teaching?” This question was investigated using multiple regression, as shown in the Table 17.

**Table 17:** Multiple regression analysis.

Dependent variable: implementation of e-learning in their teaching					
Demographic variables	*B*	*t*	Sig.	Tolerance	VIF
(Constant)	3.908	12.64	0.000^***^		
Gender	–0.036	–1.661	0.099	0.896	1.117
Years of teaching experience	–0.137	–2.807	0.006^***^	0.792	1.263
Technology skills level	0.094	3.053	0.003^***^	0.924	1.082
Technology-training programs	0.093	2.157	0.033^**^	0.959	1.043
Academic department	0.032	2.638	0.009^***^	0.964	1.038
Access to the internet	0.011	3.375	0.001^***^	0.980	1.020
Adj. *R*^2^	55.6%				
*R* ^2^	57.8%				
*F*	6.086				
Sig	0.000				
Durbin-Watson	1.872				

A multiple regression analysis was conducted to see how the six demographic factors (gender, academic department, years of teaching experience, technological skills level, technology-training programs, and internet connection) predicted e-learning implementation among Yarmouk University faculty members. The variance inflation factor VIF and the tolerance test were used to assess multicollinearity. The results reveal that all variable values were less than 10 in the VIF test and greater than 0.1 in the tolerance test, indicating that there was no difficulty with multicollinearity. As indicated in Table 17, the combination of the six demographic factors is substantially predictive of e-learning adoption among Yarmouk University faculty members, with *F* (6.085700) = *p* 0.000.

The adjusted *R*2 for the multiple regression analysis was 55.6%, suggesting that the combination of the six demographic factors employed in the model can explain for roughly 55.6% of the variation in e-learning adoption at Yarmouk University.

## 5. Discussion and conclusions

### 5.1. Discussion of research questions

#### 5.1.1. Research questions one and two

In the second part of the survey, participants were asked to rate their current level of knowledge and usage of e-learning using nine statements for knowledge and seven statements for usage. Participants rated the following items for Knowledge: (1) general distance education; (2) computerized electronic learning; (3) online learning; (4) internet learning; (5) online computing accounts; (6) e-mail programs; (7) web page creation programs; (8) web searching; and (9) online social networking service. Participants assessed the following items for usage: (1) upgrade your computer with the appropriate hardware and software for e-learning, (2) activate your computing accounts, such as Blackboard, Vista, WebCT, and so (3) administer my classes (e.g., blackboard: publish homework or other class requirements, grades, project information, or ideas), (4) share my students’ work on the Internet, and (5) encourage learning and research (e.g., use content-specific tools), (6) collaborate with colleagues and/or other professionals; and (7) interact with students outside of class.

According to the findings, the general level of understanding of e-learning among Yarmouk University faculty members was extremely good, with a mean 3.049 on a 4-point Likert scale (SD = 0.541). Furthermore, the general amount of Yarmouk University faculty members’ use of e-learning was often with a mean 3.640 on a 5-point Likert scale (SD = 0.756).

Contrary to predictions, the findings of the knowledge and usage levels show that the majority of faculty members have an excellent understanding of e-learning and use it in their teaching. The findings of this study do not support to some degree the prior literature which stated that e-learning in higher education in developing countries is still in its infancy stage and there are still various hurdles that limit successful integration for it in higher education.

There are several interpretations for these observations. One of them is that this study used a self-report to acquire information from participants. Self-reporting is one of the most common ways for people to express their opinions or activities. While there are some benefits to utilize self-reports to measure the opinion or behavior of others, several restrictions remain. A common issue in self-reporting is that people sometimes feel partial when they reveal their thoughts or behaviors. Individuals, for example, will react more frequently to perceive their observations or ideas in a more desired and suitable light, either consciously or subconsciously (McDonald, 2008). This might explain the responses to the question, demonstrating a high level of awareness and use of e-learning in teaching by faculty members.

However, there is another possible explanation: these findings may be a true representation of the reality that e-learning is at a medium to high level of awareness and utilization in Jordanian universities, especially during the pandemic where on-line learning became a “necessary good.” The high rate of e-learning adoption among faculty members may be attributed to the fact that e-learning began in The MOHE has conducted various creative programs aiming at the deployment of e-learning and its applications in academic universities.

In 2003, MOHE created the Computer and Information Center (CIC) to provide ICT services to schools and academic institutes. MOHE launched a programming initiative in 2008, followed by a network project for classrooms. These efforts sought to connect schools and directorates over a large network. MOHE has performed various endeavors in collaboration with local and foreign corporations, such as Cisco, to build an interactive version of curricula for all government K-12 schools, as well as other educational tools, such as the multimedia library and the electronic class structure. In view of the government’s attempts to encourage technology use in education, numerous Jordanian institutions typically establish initiatives to offer suitable (ICT) infrastructure and electronic learning materials for students in higher education. Many Jordanian universities, including Jordan University (JU), Yarmouk University (YU), Al-Balk’aa University, Al-Hussin University, Prince Summaya University (PSU), and Aqaba University, have official agreements with Cisco to include e-learning services in their curriculum (Al-Jedaiah, 2020). Her Majesty Queen Rania Abdullah funded Edraak, an open internet platform, in 2013. It provides free access to educational materials and more than 100 online courses in Arabic and English in subjects such as health, science, and industrial management. Jordan University of Science and Technology (JUST) established an e-learning & remote learning section in 2013 to support university academics in developing online courses and to deliver 21st century learning to over 60,000 students (Al-Jedaiah, 2020).

Another probable explanation is the condition that the entire planet was in struggling through the pandemic, during which e-learning technologies played a critical role in education. The pandemic forced educational institutions to swiftly transition to distant and online learning. As a result of the globe’s emergency situation, universities all around the world adopted online learning as an alternative to face-to-face education. Jordan was one of the first countries in that responded to the crisis by mandating a closure of all educational institutions. The Ministry of Education switched to distant learning technologies which were at certain points the sole alternative to continue learning during the outbreak. This sole choice has compelled universities including faculty members to learn and use e-learning tools.

#### 5.1.2. Research question three

*What are the primary challenges that can impede faculty members at Yarmouk University from properly using e-learning in their teaching?*

Faculty members’ responses on the Policy and Support measure ranged from 3.357 to 3.707. The overall evaluation of policy impediments and support from Yarmouk University faculty members indicated that they received mid-level policy support for the use of e-learning (*M* = 3.567, SD = 0.594) to an indeterminate degree. The data indicated that faculty members had difficulties while integrating e-learning. The primary issues are that faculty members are not involved in developing strategic plans to deploy e-learning in teaching, and there is no follow-up from specialists during e-learning in teaching use. Although faculty members play an important role in this process, they are unsure if the institution and ministry have adequate strategies and funds to help them in implementing e-learning in their teaching. Furthermore, the findings revealed that there are infrastructure and resource limitations that faculty members encounter while implementing e-learning. The most significant of these impediments were a lack of sufficient fast Internet connections and a dearth of digitized texts for the majority of curriculum. However, faculty members were dissatisfied with the technological assistance provided to them when adopting e-learning.

Despite the obstacles that faculty members experienced, their responses to the Attitude toward e-learning Implementation suggested that they had a favorable and high degree of attitudes toward the implementation (*M* = 3.91, SD = 0.59). According to the averages, faculty members would want more time to prepare activities/lessons that incorporate information technology, and they would like to see more supportive attitudes from department chairmen about utilizing e-learning. Furthermore, faculty members expressed high levels of satisfaction with the university’s preparation and development settings for e-learning implementation (*M* = 3.66, SD = 0.67). The averages revealed that they did receive adequate high quality in-service e-learning development programs on how to apply e-learning, and they were not given enough time to gain skills enabling them to execute e-learning.

The findings revealed that Jordanian institutions in general, and Yarmouk University in particular, have several problems that limit efficient implementation of e-learning. The majority of these issues are associated with policy, resources, and infrastructure. These findings were predicted by the Jordanian setting and the literature review.

Jordan is a small country with a small population and little resources. As a result, the Jordanian economy is modest in comparison to the economies of other Arab nations, particularly surrounding countries, due to limited supplies of water, oil, and other natural resources, which explains the government’s reliance on foreign aid. In addition, there are additional economic issues that the Jordanian government faces, such as chronic high unemployment rates, underemployment, budget and current account deficits, and government debt. Jordanian higher education is influenced by a variety of circumstances, including the country’s economic position and the region’s political climate. In his recent study, Osman (2018) addressed the most significant factors affecting education in Jordan, specifically higher education, and these were conservative cultures, a lack of legal frameworks, a lack of financial resources, untrained employees, traditional teaching methods, and a lack of infrastructure for the adoption and improvement of e-learning. Furthermore, Jordan’s educational systems are under severe strain as a result of the flood of Arab refugees fleeing civil conflicts in neighboring countries, making it difficult to implement new technology and tactics to provide excellent education for all (Jalbout and Farah, 2016).

Costs in Jordan are still trailing behind and are regarded one of the difficulties to integrating e-learning in higher education in Jordan successfully. According to Jordan has 87% internet penetration compared to Belgium’s 94.9%, Denmark’s 96.9%, Estonia’s 97.9%, Germany’s 96.2%, and Canada’s 91%. The numbers shown above indicate an acceptable communication infrastructure in which a sizable population is classified as technology users. However, Alshammari et al. (2016) noted that although communication technology nowadays is becoming highly inventive and available all over the world including the Middle East but the application of information and communication technology is still limited. The reasons for this low rate of adoption may be related to the availability of these technologies. As a result, there is a severe problem with the expenses of building e-learning systems in developing nations, even though they are required, and that it is essential not only to identify the system, which is already costly, but also to construct and maintain this system (Bringula and Basa, 2011).

While Jordan has an 87% internet penetration rate, internet access remains a significant barrier to e-learning deployment in Jordan. Alkhawaja and Halim (2019) stated in their study that there are still numerous problems associated with the use of e-learning in higher education in Jordan. These hurdles include technological impediments caused by limited bandwidth or poor internet access, as well as difficulty in transitioning from traditional classrooms to course management systems. In light of the COVID-19 pandemic, the globe relies on the internet as the sole method and means of doing remote learning. In this context, internet connection issues, whether of availability, quality, or cost, have emerged as major hurdles that prohibit the continuance of the sole learning technique accessible in this situation. According to Alkhawaja and Halim (2019), the challenges linked with Internet connectivity are diverse. Although internet access is available in most regions, this does not always mean that everyone can afford it due to its high cost in comparison to the typical Jordanian household income.

Many studies agree with Yarmouk University faculty members on the level of curriculum that digital texts are not accessible for (e.g., Almaiah et al., 2020). The issues which are associated with e-learning system curriculum reach deeper than just the question of availability of digital textbooks. Al-Hawamdeh (2011) investigated the curricula barriers that may impede the use of an e-learning system and discovered a number of them, including: not focusing the objectives of traditional courses on e-learning, a lack of educational activities supporting the use of e-learning, course length and subject density, difficulty converting academic courses to electronic software, and the nature of traditional subjects included in the course does not cope with modern technology. This demonstrates the enormity of the undertaking of integrating e-learning. Equipping universities with the tools and modern equipment required for e-learning, as well as the operating and maintenance costs and the cost of producing the software required for the educational process, is a real and unique challenge for a country with limited resources, such as Jordan, where universities frequently rely on university tuition paid by students and government funding.

Despite the difficulties that faculty members demonstrated, they had good and high attitudes toward implementation. They also expressed a high level of satisfaction with the university’s preparation and development settings for e-learning deployment. These findings defied expectations, according to earlier research. The explanation for these unexpected results returns to the large efforts and settings that the Jordanian government is making in terms of e-learning adoption at higher education institutions in general, as discussed in the two questions, and to the large efforts that Yarmouk University is making in the way of creating an effective e-learning system in particular. In 2018, Yarmouk University launched e-learning center that sought to foster and disseminate the culture of e-learning *via* the internet among academics, staff and students. The center employs cutting-edge methods and technology to train, equip, and empower faculty members to produce high-quality electronic content that meets international standards, as well as to fully support the four main components of online e-learning, which are faculty members and students, learning content, assessment methods, and electronic communication. The most important tasks it performs are assisting faculty members in designing instructional content for the e-learning platform and designing examinations for various exam platforms, resolving technical issues that students and faculty members face, and providing periodic guidance on the use of educational platforms and programs (Yarmouk University, 2021).

Another explanation for faculty members’ favorable sentiments might be changes in online certification procedures implemented by the Directorate of Recognition and Equivalency of Certificates (DREC), which is linked with the Ministry of Higher Education and Scientific Research. When remote learning began in higher education in Jordan in 2003, there were strong limits on online certificates. At the time, the ministry had just recently recognized or equated online path (non-traditional) credentials with in-person ones, and most (if not all) colleges had not admitted students pursuing full or partial online route studies. As a result, learners through the remote learning policy suffered from not having their credentials recognized, with many organizations and institutions refusing to hire individuals with a degree obtained through distance learning. This event significantly influenced unfavorable attitudes and confidence issues with e-learning pathways. Initiatives in conferences began in 2011 to review the non-recognition of non-traditional credentials to be acknowledged and equated. In 2012, Ministry of Higher Education and Scientific Research began to recognize institutions that provide non-traditional education, including electronic, blended, distance and open education, according to new instructions and principles (Ministry of Higher Education and Scientific Research, 2021). This move has broadened the possibilities for studying and teaching in Jordan.

#### 5.1.3. Research question four

*Is there a difference in e-learning knowledge, use, and hurdles between male and female faculty members at Yarmouk University?*

The findings revealed no significant differences between male and female faculty members in terms of knowledge, use, or impediments encountered. The literature review included several research that confirmed the aforementioned conclusion, but there were also numerous studies that claimed that there is a difference between male and female attitudes toward information technology, including e-learning, as described in the literate review. However, given the Jordanian context, the discovery was unexpected. In Jordan, where conventional expectations and cultural constraints continue to hamper women’s development (United States Agency for International Development, 2021), women suffer a large gap between their constitutional rights and accepted societal standards. According to statistics, Jordanian women’s economic participation rate is among the lowest in the world, with less than a fifth of women in the country working (United States Agency for International Development, 2021). Yarmouk University is located in the center of a small city surrounded by many villages and rural areas where customs and traditions are much more rooted than in big cities, and the majority of the university population, whether students or staff, are from the surrounding areas where provisions of customs and traditions occupy a significant place in people’s lives and influence their decisions. This is why the study’s gender variable was supposed to be investigated. There are initiatives leaded by the USAID which aspires in all of its projects in Jordan to minimize gender inequities, empower women and girls to recognize their rights and determine their own objectives in life, and assist Jordan establish a future of economic stability and self-reliance. Hopefully, the outcomes of this section are a decent sign of their efforts.

#### 5.1.4. Research question five

*Are the specified demographic characteristics of faculty members (academic department, years of teaching experience, degree of technological skills, technology-training programs, internet connection) connected to the incorporation of e-learning in their teaching?*

The findings revealed that all five factors were related to faculty members using e-learning into their teaching. The researcher feels that these findings are predictable and logical.

The rationale for evaluating the influence of the academic department of faculty members for the academic department variable pertains to the difference in technological settings from college (or faculty) to college. While one college has computer and internet facilities, as well as a computer linked to the internet for each faculty member, other colleges in the same institution have a relatively limited number of computers. Furthermore, the academic department variable reflects to some extent the difference in faculty member specialties, as some of them may have studied courses in the application of computer and internet as part of their majors, whereas other faculty members did not study any courses on technologies. The findings of this study revealed that there was a positive effect on the academic departments to which faculty members belonged when it came to implementing e-learning, with computer science, economics, engineering, education, and law faculty members having the most influence in implementing e-learning in their teaching. The researcher believes there are two possible explanations for the findings.

The first explanation is that these results are the result of individual efforts by faculty members from the most influential academic departments (computer science, economics, engineering, education, and law), or that the results were referred to the significant difference for these academic departments, which assisted their faculty members in applying e-learning in teaching, and that in order to stand on these differences, the situation of academic departments should be addressed in detail. To some extent, the findings agreed with Al-Qudah and Maqableh (2014), who found that academic department has a significant impact on the most challenges that faculty members face when implementing e-learning in their teaching at private universities in Jordan, and the challenges were greater for faculty members from humanitarian academic departments than faculty members from scientific academic departments. The researchers said that because scientific areas are more exposed to computers and internet applications than humanities, it is predicted that faculty members in scientific departments will have significantly superior technology abilities than their counterparts in humanitarian departments. This finding contradicted Badarneh et al. (2016), who concluded that there were no statistically significant differences in the degree of competencies (computer use competencies, network and Internet use competencies, e-learning culture competencies) held by faculty members at one of Jordan’s universities (Al-Balqa Applied University) in all areas of e-learning based on the academic department variable.

In terms of the years of teaching experience variable, the results revealed that younger faculty members were better equipped to incorporate e-learning into their teaching. According to the researcher, younger professors are more willing to use electronic technologies, and those with less experience are more receptive to technology, so they are more receptive to experimenting and introducing new things to their classes, and more convinced of the need to change the old methods of education. This study corresponds with Badarneh et al. (2016) which revealed that the competences of the e-learning culture were greater among instructors with less than 5 years of experience than instructors who were between 5 and 10 years in experience. This result, however, contradicted the findings of Al-Qudah and Maqableh (2014), who discovered that the years of experience variable has no statistical significance on the e-learning challenges (technical challenges, financial and administrative challenges, and professional challenges) that face faculty members in private Jordanian universities. The explanation for this, according to the researchers, might be related to the novelty of e-learning in Jordanian institutions, particularly in private Jordanian universities, which minimizes the influence of the years of experience variable. Technology’s availability does not imply its usage, because people who use it must see a clear advantage in order to use and adopt it enthusiastically.

The results for both factors demonstrated that there is a beneficial influence for technology-training programs and technology skills level on faculty members utilizing e-learning in their teaching. These findings were anticipated by the researcher. These outcomes are reasonable and make sense, according to the Technology Acceptance Model (TAM). Technology-training programs and advanced technology skills boost faculty members’ expertise with technology, which affects their impression of technology’s ease of use favorably. This view, in turn, generates favorable attitudes toward technology, good intentions to utilize it, and, as a result, a positive degree of usage of technology.

## Data availability statement

The original contributions presented in the study are included in the article/supplementary material, further inquiries can be directed to the corresponding author.

## Ethics statement

The studies involving human participants were reviewed and approved by Kansas University Ethics Board. The patients/participants provided their written informed consent to participate in this study.

## Author contributions

AA designed the research and collected data. HA and MA-D helped with the analysis of the results. RA proofread and edited the article. All authors contributed to the article and approved the submitted version.

## Conflict of interest

The authors declare that the research was conducted in the absence of any commercial or financial relationships that could be construed as a potential conflict of interest.

## Publisher’s note

All claims expressed in this article are solely those of the authors and do not necessarily represent those of their affiliated organizations, or those of the publisher, the editors and the reviewers. Any product that may be evaluated in this article, or claim that may be made by its manufacturer, is not guaranteed or endorsed by the publisher.
